# An Overlooked Mandibular-Rubbing Behavior Used during Recruitment by the African Weaver Ant, *Oecophylla longinoda*

**DOI:** 10.1371/journal.pone.0008957

**Published:** 2010-02-01

**Authors:** Olivier Roux, Johan Billen, Jérôme Orivel, Alain Dejean

**Affiliations:** 1 Écologie des Forêts de Guyane, Centre National de la Recherche Scientifique: Unité Mixte de Recherche, Kourou, France; 2 Laboratory of Entomology, Zoological Institute University of Leuven, Leuven, Belgium; 3 Laboratoire Évolution et Diversité Biologique, Centre National de la Recherche Scientifique: Unité Mixte de Recherche, Toulouse, France; University of Bristol, United Kingdom

## Abstract

In *Oecophylla*, an ant genus comprising two territorially dominant arboreal species, workers are known to (1) use anal spots to mark their territories, (2) drag their gaster along the substrate to deposit short-range recruitment trails, and (3) drag the extruded rectal gland along the substrate to deposit the trails used in long-range recruitment. Here we study an overlooked but important marking behavior in which *O. longinoda* workers first rub the underside of their mandibles onto the substrate, and then—in a surprising posture—tilt their head and also rub the upper side of their mandibles. We demonstrate that this behavior is used to recruit nestmates. Its frequency varies with the rate at which a new territory, a sugary food source, a prey item, or an alien ant are discovered. Microscopy analyses showed that both the upper side and the underside of the mandibles possess pores linked to secretory glands. So, by rubbing their mandibles onto the substrate, the workers probably spread a secretion from these glands that is involved in nestmate recruitment.

## Introduction

The canopies of tropical forests and tree crop plantations are occupied by “territorially-dominant” arboreal ant species that defend absolute spatial territories, usually from colonies of other dominant species, including conspecifics [1], [2]. These species are characterized by populous colonies, the ability to build large and/or polydomous nests, and a highly developed intra- as well as inter-specific territoriality that causes their territories to be distributed in a mosaic pattern in the canopies [1], [2].

The workers of territorially-dominant arboreal ants deposit landmarks on their territories, something first shown in *Oecophylla*, a genus represented by two species, *O. longinoda* and *O. smaragdina*, from Africa and Australasia, respectively. *Oecophylla* landmarks are visible, brownish anal spots containing true territorial pheromones that delimit their territories from those of neighboring colonies [3]–[5]. These landmarks are persistent, lasting for more than a year [6], [7], and are recognized by other ants that adapt their behavior so as to avoid encountering the occupying ants [8]; fighting only occurs when growing colonies expand their territories past these landmarks [2]. Furthermore, *Oecophylla* marks are perceived and used as kairomones by Lycaenid caterpillars [9].

All of the territorially-dominant arboreal ants studied exhibit a very efficient predatory behavior based on group ambush and the spread-eagling of prey that, in addition to their territoriality, provides good protection to their host trees [2]. Consequently, they have been used as biological control agents; *O. smaragdina*, in particular, was already used in ancient China [10]. Territorially-dominant arboreal ants not only directly protect their host trees from arthropod herbivores by preying on them, but also by disturbing them (trait-mediated indirect interaction) [11], [12]; for example, fruit flies and chrysomelid females avoid laying eggs on plants when they perceive *Oecophylla* landmarks [13]–[16].

**Figure 1:**
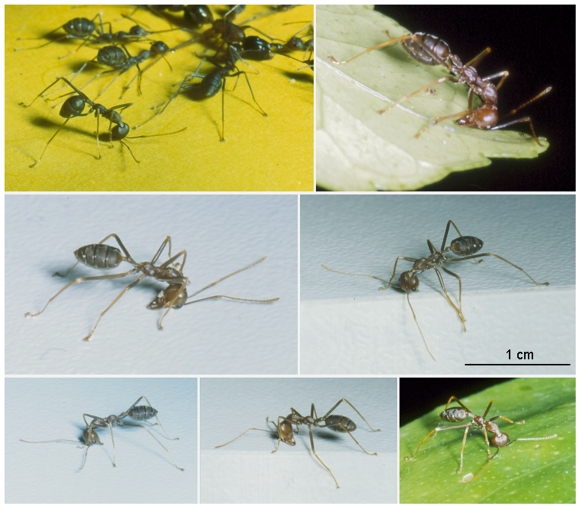
Illustrations of the posture of marking workers. Includes a case where a worker is marking while nestmates are spread-eagling a *Camponotus* worker (upper left photo).

Therefore, the landmarks deposited by *Oecophylla* affect competing ant colonies, facilitate mutualistic and parasitic activities, and deter herbivorous insects. While rearing *O. longinoda* colonies, we noted each time that we provisioned them that some workers (both majors and minors) often rubbed the Petri dish containing the food first with the underside of their mandibles and then with the upper side in a surprising posture (Fig. 1). Sometimes they only rubbed the underside. We hypothesized that this might correspond to a new kind of marking behavior used to locally inform nestmates of a new event on the territory. We therefore conducted a series of experiments to verify if this behavior is triggered when the ants discover (1) a new territory (the Petri dish), (2) prey (frozen crickets furnished during the experiment), or (3) even alien ants. We then looked for the presence of glands thought to be responsible for the secretion of compounds during this behavior.

## Methods

### Ethics Statement

This study was conducted according to relevant national and international guidelines.

### Rearing Ant Colonies

This study was conducted on six ant colonies each constituted of more than 1,000 workers and their queen collected from isolated trees in Cameroon. The colonies were installed on potted *Citrus limon* trees in France two days after being collected from the field and kept for several months or years in the laboratory. The pots were placed on tables (80×140 cm), the feet of which were placed into receptacles containing water to keep the ants from escaping. The colonies were fed with honey and prey (cricket larvae) deposited in a Petri dish on the table. Experiments were conducted 2 weeks after the colonies were installed and after they had had the time to mark their territories.

### Behavioral Tests

Two experimental designs were used. We first explored the intensity of the “new” mandibular marking behavior as a function of the food resource or alien ant discovered by the workers. In the territory belonging to the ant colonies (the tables), the behavior of the workers was noted after they discovered a drop of honey (n = 40), a prey (a cricket larva, *Acheta domesticus*, n = 40), or a *Camponotus* sp. worker (n = 30). The different items were deposited at least one meter away from the potted tree and the lowest subnest was at ca. 60 cm in height in the foliage of the trees. For each replicate, the tested item was deposited at a different place on the table. The number of workers using the mandibular marking behavior (henceforth “marking workers”) and the total number of mandibular marking behaviors (henceforth “marking”) produced were recorded during 10 minutes before and 10 minutes after the introduction of the food resource or alien ant.

Secondly, we explored the effect of the discovery of a new territory and then the discovery of a prey. For this, a clean circular piece of paper, 29 cm in diameter, was placed onto the territories of the ant colonies as previously described and left for two days to allow the workers to appropriate this new territory. The first experiment consisted in superimposing a new, clean piece of paper a quarter size of the original circle (test: “new, unmarked” area); the three other quarters served as a control (so, three times the surface area of the tested area). We noted the number of times the ants used the mandibular marking behavior on these two areas during 10 minutes after the introduction of the new, unmarked area (n = 35). At the end of these 10 minutes, a prey (*A. domesticus*) was carefully deposited in the center of the “circle”; we then again noted the number times the ants used the mandibular marking behavior during 10 minutes after the introduction of the prey (n = 35). We also noted the number of workers visiting the entire circle during the 10 minutes before the introduction of the clean piece of paper, the 10 minutes that followed this introduction, and during the 10 minutes after the introduction of a prey.

During the two experiments, the workers were already accustomed to their rearing conditions in the laboratory. Like in natural conditions, they move very slowly and their behavior is very easy to observe. The observers only had to enter a mark on a grid sheet (with 10 horizontal lines each corresponding to an eventual marking worker as during preliminary experiments we noted that it is very exceptional that more than 6 workers marked during the 10 minute period of time) each time a worker used the mandibular marking behavior. The number of times that the workers marked likely reflected their level of stimulation. Nevertheless, with the aim of avoiding direct recruitment outside of this area, we removed the ants that left the surroundings of the experimental area (at a distance of ca. 20 cm). These ants were reintroduced into their nests after each experiment (only one experiment per day and one experimental set-up).

### Microscopy Analyses

We used light microscopy on semi-thin sections (1 µm) as well as scanning electron microscopy (SEM) to search for glands involved in the production of an eventual marking pheromone. We used the same methods as Schoeters and Billen [17] for light microscopy; for the SEM, the heads of the ants were sputter coated with gold and viewed through a JEOL JSM-6360 scanning microscope.

### Statistical Analyses

In the first series of experiments, we compared both the number of workers that marked and the number of markings before and after the introduction of honey, prey or an alien ant. We also compared differences in the marking behavior between these introduced elements. In the second series of experiments, we compared the number of markings on the new piece of paper (new territory; 1/4 of a circle) with the number on the control (3/4 of a circle) after (1) the introduction of the clean piece of paper and (2) the introduction of the prey. For both the new piece of paper and the prey, theoretical values were obtained by multiplying the core values of the new territory by three. We also compared the number of workers on the entire circle before the experiment and after the introduction of the clean quarter piece of paper and then the introduction of the prey.

Because our data were highly structured due to the number of colonies (6) used repeatedly (5 to 7 times each), we used the Generalized Linear Mixed Model (GLMM) on R 2.8.1 (R Development Core Team, 2008) with the “glmer” function of the “lme4” package by Bates and Maechler. The GLMM was run on counts (Poisson distribution option) for all comparisons, using treatments (i.e., the introduction of the different items: a clean piece of paper, honey, prey or ants) as a fixed effect, colonies as a random effect and replicates as a nested random effect in the colony factor.

## Results

### The Workers' Behavior and Behavioral Tests

When marking, the workers first rub the underside of their mandibles onto the substrate in a series of side-to-side movements, with the tips of mandibles describing an arc. Next, they tilt their head to one side and pivot it around, bringing the tips of their mandibles under their thorax. Then, they rub the upper side of their mandibles using the same side-to-side movements that they used to rub the underside (Fig. 1). The complete marking behavior takes approximately 5 seconds and can be repeated several times by the same ant (i.e., up to 30 times in 10 minutes after discovering a prey; data not shown).

**Figure 2:**
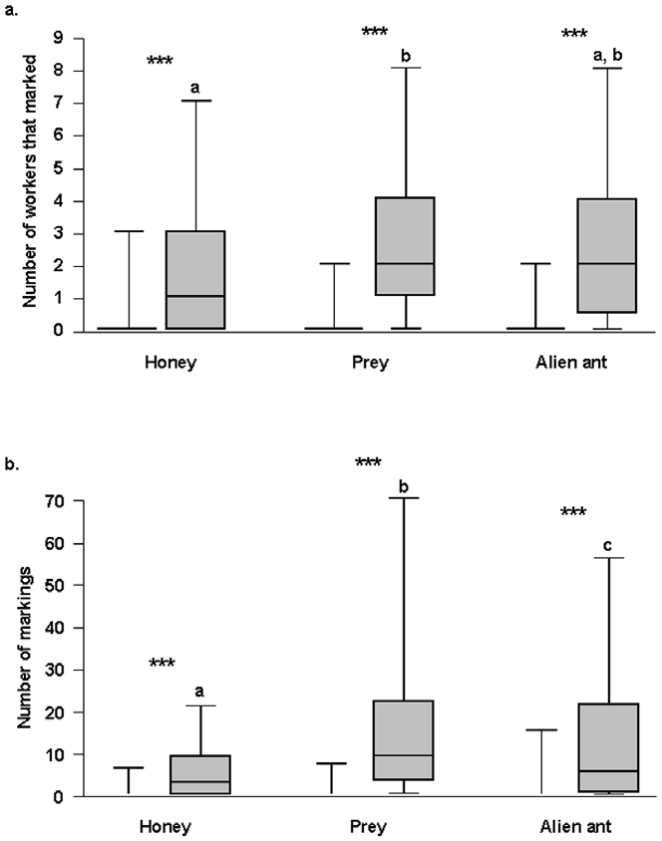
Effects of the introduction of honey, prey, or alien ants on the intensity of the marking behavior. Number of workers that marked territories (A) and number of markings (B) 10 minutes before (left) and 10 minutes after (right) the introduction of honey, prey or alien ants. The box plots indicate the median (wide horizontal bars), the 25th and 75th percentiles (squares), and the minimum and maximum values (whiskers). Statistical comparisons 10 minutes before (left bars) and 10 minutes after (right bars) the introduction of honey, prey or an alien individual were made using glmm; *** = P<0.001. Comparisons between treatments after the introduction of food items were made using glmm (different letters indicate significant differences at least at P<0.01).

During the first series of experiments we noted that the number of marking workers increased significantly after we deposited honey, prey or alien ants on the tested area (z = 5.303, 6.536 and 5.555, respectively; P<0.001 in all cases); (Fig. 2A). The number of markings followed the same pattern (z = 9.258, 13.613 and 13.254, respectively; P<0.001 in all cases); (Fig. 2B). Furthermore, the introduction of prey resulted in a greater number of workers that mark than did the introduction of honey (z = 3.154; P = 0.0016), while the comparisons between honey and alien ants, or prey and alien ants resulted in non-significant differences (z = −1.757 and 1.219, respectively); (Fig. 2A). The number of times the workers marked was, however, significantly different in each case with a greater difference between honey and an alien ant and honey and a prey (z = −9.784 and 12.797, respectively; P<0.001 in both cases) than between an alien ant and a prey (z = 2.574; P = 0.01) (Fig. 2B). Note that the number of markings per worker increased significantly after we deposited honey, prey or alien ants on the tested area (z = 6.094, 8.452 and 7.473, respectively; P<0.001 in all cases).

**Figure 3:**
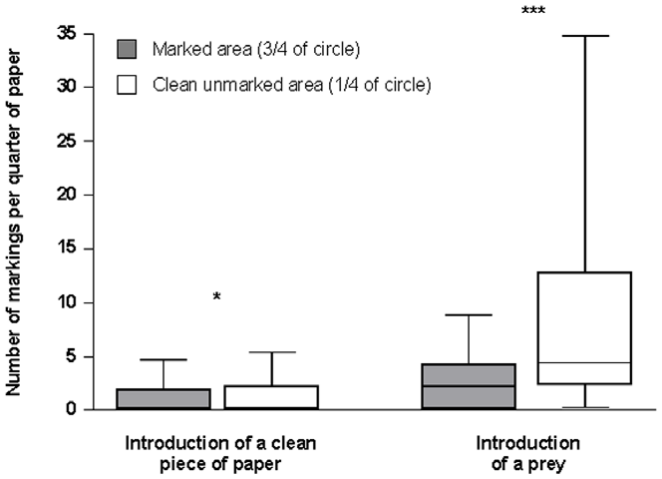
Number of markings per quarter surface unit when a clean piece of paper and then a prey were introduced. We compared the number of markings on two areas (a marked area or ¾ of a circle 29 cm in diameter and clean; unmarked areas or ¼ of the same circle) during 10 minutes after the introduction of the new, unmarked area (n = 35). Just after, a prey was carefully deposited in the centre and we again compared the number of markings on the two areas during 10 minutes. The box plots indicate the median (large horizontal bars), the 25th and 75th percentiles (squares), and the minimum and maximum values (whiskers). Statistical comparisons were conducted using glmm; *** = P<0.001 and * = P<0.05.

**Figure 4:**
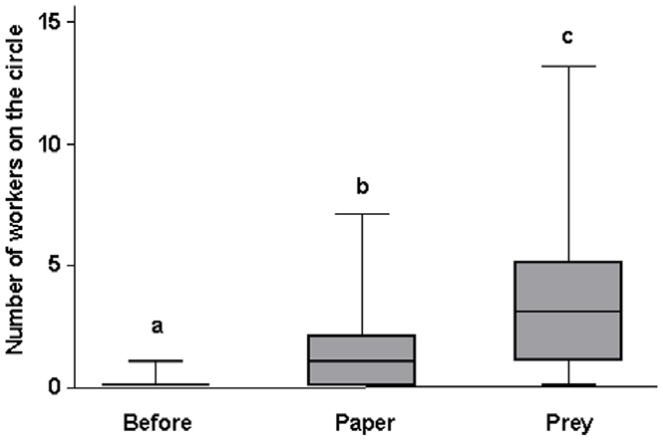
Number of workers visiting the circle before the introduction of clean paper, and when clean paper and then a prey were introduced. We compared the number of workers on the entire circle during 10 minutes before the experiment, during 10 minutes after the introduction of the new, unmarked area and then 10 minutes after a prey was carefully deposited in the centre of that area. The box plots indicate the median (large horizontal bars), the 25th and 75th percentiles (squares), and the minimum and maximum values (whiskers). Statistical comparisons were conducted using glmm; *** = P<0.001.

During the second series of experiments, the number of markings on the clean pieces of paper (corresponding to the discovery of a new territory, without markings) was proportionately significantly higher than the number of markings on the control area (1) during the 10 minutes following the introduction of the clean pieces of paper (the difference in surface area being taken into consideration, z = −2.024; P<0.05), and (2) after a prey was introduced (z = −16.343; P<0.001) (Fig. 3). Meanwhile, during the entire duration of the experiment (the three series of 10 minute controls) the number of workers had significantly increased over the entire circle between the different stages of the experiment: before and after the introduction of a clean piece of paper (z = 3.736; P<0.001), and before and after the introduction of a prey (z = 6.524; P<0.001) (Fig. 4). During these experiments, we did not note workers dragging their gaster on the substrate (with or without extruded rectal glands) to recruit nestmates at short- or long range, probably because we removed all of those that left the surroundings of the experimental area and moved at least ca. 20 cm away.

### Microscopy Analyses

**Figure 5:**
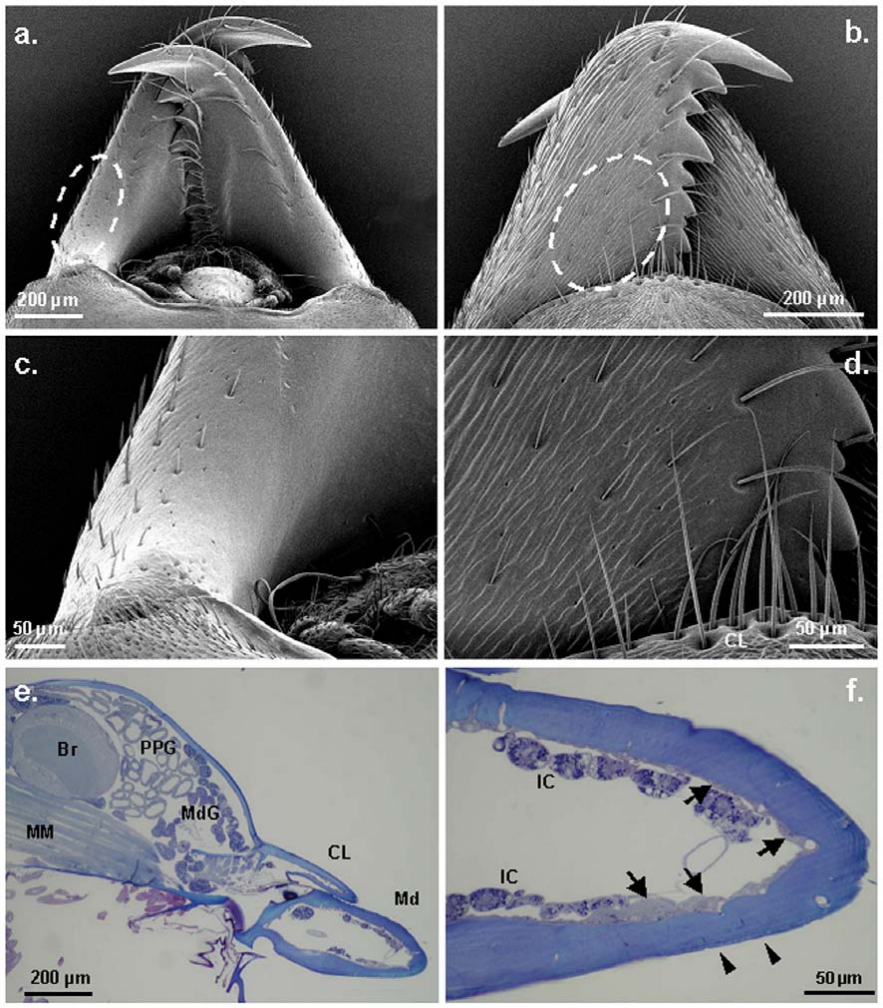
Illustration of the presence of intramandibular glands in *Oecophylla longinoda* major workers. A. The underside and B. upper side of the mandibles. Areas indicated by dotted ovals show the location of pores; C. Close-up of the proximal part of the underside and D. upper side of a mandible (A–D: scanning electron microscopy); E. and F. (light microscopy). Thin, longitudinal sections of the head and a mandible showing the location of the intramandibular gland cells (arrows), and ducts (arrowheads). Br: brain, CL: clypeus, IC: intramandibular cells (fat cells and oenocytes), Md: mandible, MdG: mandibular glands, MM: Mandibular muscle, PPG: postpharyngeal gland.

SEM observation showed the presence of pores on both the under- and upper sides of the mandibles (Fig. 5). They have a diameter of about 0.5–1 µm. On the underside of the mandibles, the pores are mainly located near the proximal external edge (Fig. 5A, C); whereas, on the upper side, they occur rather near the proximal internal edge (Fig. 5B, D). Semi-thin sections confirmed the presence of gland cells near both the under- and upper sides of the mandibles (Fig. 5E, F). These glands are formed by bicellular units, each consisting of a single secretory cell and its associated duct cell, which corresponds to class 3 glands in the classification by Noirot and Quennedey [18]. Each secretory unit opens directly to the outside through the cuticle at an oblique angle and pointing towards the tip of the mandible.

## Discussion

We have demonstrated that the discovery of a new territory, a sugary food source, a prey or an alien ant triggers a peculiar behavior in *Oecophylla* workers that first rub the underside of their mandibles onto the substrate, and then tilt their head to rub the upper side as well. This behavior complements other, already-known marking behaviors in *Oecophylla* workers such as (1) using anal spots containing a territorial pheromone as territorial markers, (2) dragging the gaster so that the sternal gland openings come into contact with the substrate so as to deposit short-range recruitment trails, and (3) dragging the extruded rectal gland onto the substrate to deposit the trails used in long-range recruitment to food, new territory or enemies [4].

Visual cues may have interfered with our experiments when we superimposed the clean piece of paper or when we introduced the prey. Indeed, *O. longinoda* workers use group ambush hunting where preys are detected by sight. Also, when an individual successfully seizes a prey, it takes up a typical posture: it lifts its gaster. This is a visual signal (probably complemented by the emission of an alarm pheromone from the mandibular gland, see [19], [20]) that permits it to attract nestmates situated in the vicinity during group ambush hunting where several workers are situated very close to each other [21], [22]. Also, the posture of the workers during mandibular marking could be a visually attractive stimulus complemented by the use of chemicals. This marking behavior seems to correspond to a new kind of short-range recruitment as the number of workers increased in the focal circle. In already-known cases of short-range recruitment involving territorial marking (including in *Oecophylla*), workers run in short, looping circles while dragging their gaster onto the substrate and attract nestmates situated in a radius of 10 to 30 cm [4], [23]–[26].

Another kind of short-range recruitment was described in *O. longinoda* major workers that emit mandibular gland secretions [19], [20]. Gaz chromatographs of their mandibular glands indicated the presence of at least 33 chemicals that trigger a gradual series of responses as the ants approach the source of the emission of the chemicals. By presenting pure samples of the principal components to foraging workers, it was demonstrated that hexanal triggers an alarm in workers, while a sample of 1-hexanol attracts them from a radius of ca. 10 cm (but repelled them when they are at only a few mm away from the source). Finally 3-undecanone and 2-butyl-2-octenal induces attracted workers to bite any alien object in the vicinity [19], [20]. This is followed by pulling backwards, so that prey or alien ants are spread-eagled when several nestmates are attracted; see [21]. Therefore, we cannot completely exclude that during our study on the effect of mandibular marking behavior the mandibular gland also emitted secretions. Nevertheless, this emission generally occurs during the actual discovery of a prey or an alien ant, while the mandibular marking occurs later. It is also very unlikely that the mandibular gland emitted a secretion during the discovery of a new territory. Furthermore, minor workers can be involved in marking behavior and are attracked by it, while they are repelled by the mandibular gland secretions from major workers. The composition of the secretion from their mandibular glands is very different than that of major individuals, the main components being nerol and geraniol [19], [20].

As a result, mandibular marking behavior seems to attract nestmates to a zone where their presence is necessary (e.g. new territory, food source or alien ant presence), so that the marks deposited complement the action of territorial marking and/or recruitment pheromones.

Note that the mandibular marking behavior was more intense when a prey item (a source of protein) rather than a sugary resource was discovered. This is an argument in favor of the theory that this type of marking may help to signal an unpredictable and ephemeral resource or alien ant presence. Indeed, contrary to prey, in natural conditions sugary resources are rather “permanent”, supplied by extrafloral nectaries or the honeydew produced by hemipterans attended by the workers in a favorable area [2], [22].

The presence of intramandibular glands has already been described in several ants, but their function remains unknown [17]. Whereas these glands usually open through the upper surface of the mandible only, *O. longinoda* has them both on the upper and lower sides, which makes them a very likely candidate for the rubbing behavior here described. The position of the head during this behavior matches with the position of the gland pores, as it is this lateroventral and dorsal region of the mandibles that touches the substrate during the marking. Such glands do not possess reservoirs and therefore the quantity of the secretion is probably very limited, which makes it very difficult to analyze it chemically. These low concentrations, however, apparently do not prevent the ants from marking, as they are able to spread the secretion a great number of times.

## Acknowledgments

We are grateful to An Vandoren and Alex Vrijdaghs for their assistance for the microscopy, and to Andrea Yockey-Dejean for proofreading the manuscript.
